# Volatilomics: a non-invasive technique for screening plant phenotypic traits

**DOI:** 10.1186/s13007-018-0378-4

**Published:** 2018-12-18

**Authors:** Werner Jud, J. Barbro Winkler, Bishu Niederbacher, Simon Niederbacher, Jörg-Peter Schnitzler

**Affiliations:** 1 Research Unit Environmental Simulation (EUS), Institute for Biochemical Plant Pathology, Helmholtz Zentrum München, Ingolstädter Landstraße 1, 85764 Neuherberg, Germany; 2 Present Address: Ionicon Analytic GmbH, Eduard-Bodem-Gasse 3, 6020 Innsbruck, Austria

**Keywords:** Volatilomics, Screening, Volatile organic compounds, VOC, Plant trait, Plant phenotyping, Net CO_2_ assimilation, Transpiration, Barley

## Abstract

**Background:**

Climate change represents a grand challenge for agricultural productivity. Understanding complex plant traits such as stress tolerance, disease resistance or crop yield is thus essential for breeding and the development of sustainable agriculture strategies. When screening for the most robust plant phenotypes, fast, high-throughput phenotyping represents the means of choice.

**Results:**

We have developed a plant phenotyping platform to measure the emission of volatile organic compounds (VOCs), photosynthetic gas exchange and transpiration under ambient, or abiotic and biotic stress conditions. These parameters are highly suitable markers to non-invasively and dynamically study plant growth and plant stress status, making them perfect test variables for long-term, online plant monitoring. Here we introduce the new phenotyping platform, termed VOC-SCREEN, and present results of a first case study with three barley cultivars, demonstrating that the plant’s volatilome can be successfully applied to discriminate different barley varieties.

**Conclusion:**

Volatilomics is a promising technique to non-invasively screen for plant phenotypic traits.

## Background

A phenotype is by definition the sum of all observable traits of an organism [1]. It is determined by the plant genotype (**G**), the environment (**E**) the organism is grown in, and the interaction thereof (${\mathbf{G}} \times {\mathbf{E}}$) [2]. Consequently, there is a vast variety of most diverse expressions and traits defining a phenotype. A plant phenotype includes, e.g., macroscopic and biometrically accessible traits such as leaf area, plant height, weight, shoot and root architecture, etc. Besides these apparent traits, also other, indirectly accessible properties, such as net $\text{CO}_{2}$ assimilation or transpiration rates, or the blend and amount of emitted volatile organic compounds (VOCs), are part of a plant’s phenotype.

The goal of phenotypic research is first, to reveal plant traits that can be used as phenotypic markers, and second, to develop methods to measure and quantify these traits. Eventually, these features could be used for genotype selection in plant breeding.

Over the last few decades, tremendous progress in genome sequencing has been made [3–5], both in terms of methodology and costs. The different “omics” fields,—genomics, transcriptomics, proteomics and metabolomics—have thereby dramatically improved our knowledge on the plant metabolic response to biotic and abiotic stresses, revealing new approaches for plant breeding. Simultaneously, the tremendously growing amount of genetic information demands to relate these data to the phenotypic properties of the organisms under different environmental conditions [6].

Classical plant phenotyping techniques often require destructive harvesting at specific phenological stages and are slow and costly. Modern approaches instead are favourably using non-invasive techniques, thus allowing to analyse phenotypic traits over a longer period of a plant’s development. In the last few years a lot of progress was made in gathering and evaluating the anatomic traits of different crop and model plants. Most of the lately introduced phenotyping platforms are thereby using optical measures for the qualification and quantification of plant traits [7]. With conventional RGB (red, green, blue) digital cameras, the height, leaf area and other size-related properties of plants can be determined [7–9]. More sophisticated approaches even allow to digitally reconstruct the shape of simple plants in 3D [10–12]. Fluorescence cameras allow to probe the photosynthesis II status and electron transport rates by measuring chlorophyll fluorescence of leaves [2]. Thermal imaging can be used to infer the leaf water status of a plant by measuring the temperature difference between a leaf and the surrounding air [7]. Biological basis is here the increased temperature difference when stomatal closure and reduced respiration rates lead to lower evaporative cooling of the leaves. Thermal imaging thus allows to infer the degree of a possible water deficit, or soil salinity.

Lately, some large scale root phenotyping facilities have been established, allowing to monitor root growth and root architecture in so-called rhizotron boxes [13, 14]. Again, mainly camera based systems are used to gather the raw data for the determination of these properties [14, 15].

All these imaging technologies have in common that they are producing large amounts of data which need to be processed afterwards in a possibly semi to fully automatized manner. This requires knowledge in highly sophisticated image processing technologies, which are often computer-power intensive and need to be adapted for each plant type to be investigated.

In addition to these non-invasive imaging technologies, other disruptive technologies can be used to investigate the biochemical properties (proteome and metabolome) of plant phenotypes. Chemotypes or metabotypes (metabolic phenotypes) define the unique overall metabolic fingerprint of cellular processes of genotypes under distinct environmental conditions [16, 17]. As these disruptive phenotyping technologies typically involve several sample preparation steps, they are in general slower and more labour intensive than imaging-based phenotyping approaches.

Costs and time requirements are two critical factors in large scale phenotyping. Especially in plant breeding people rely on the fast phenotyping of a large number of genotypes of a specific species under particular environmental conditions. This causes an increasing demand in high-throughput phenotyping platforms and technologies.

Our new VOC-SCREEN platform, which we introduce herein, aims to bridge the abovementioned phenotyping spheres: destructive, low-throughput genetic and chemometric (metabolomic) technologies, as well as non-invasive, high-throughput technologies. The new platform enables the analysis of the entirety of the plant’s volatile metabolites including $\text{CO}_{2}$ and water vapour, the so-called volatilome. VOCs can be used as metabolic markers to distinguish different phenotypes of not only plants, but also humans, animals, fungi and microorganisms [18–21]. Due to their eponymous physical properties VOCs are ideally suited to be measured in a non-invasive way by sampling air from an enclosure containing the test organism [22].

The VOC-SCREEN platform consists of 24 cuvettes, each enclosing potted, entire plants. Via a Proton Transfer Reaction Time-of-Flight Mass Spectrometer (PTR-ToF-MS) attached to the gas outlets of the cuvettes, the emitted VOCs can be measured in real-time [23]. The PTR-ToF-MS allows determining the plant VOC emissions or uptake in a non-targeted, quantitative way, with a mass resolution of about 4000–5000 and detection limits in the low ppt range [23] (see “Methodology” part). As this technology only allows to detect and quantify molecular mass features, the structural identity of isomers, e.g., of different mono- or sesquiterpenes, cannot be resolved. This limitation can be overcome by VOC trapping on absorption tubes followed by gas chromatography mass spectrometry (GC–MS) with a time resolution of several hours or on a daily basis [24].

Besides VOC exchange rates, the present system allows to quantify overall plant $\text{CO}_{2}$ assimilation and transpiration rates, important physiological traits linked to plant growth and plant water use efficiency [25].

We have installed the VOC-SCREEN platform in one of the phytotron chambers at the Research Unit Environmental Simulation (EUS), Helmholtz Zentrum München (HMGU) [26, 27]. However, such a phenotyping system can be installed in any controlled environment. In these chambers, environmental parameters such as temperature, relative humidity (RH), $\text{CO}_{2}$ concentration, photosynthetically active radiation (PAR), ozone ($\text{O}_{3}$), etc., are controlled in order to simulate specific environmental scenarios [25, 28]. Hence we are able to perform experiments under homogeneous, reproducible and as close as possible natural conditions. Thereby we can investigate transient effects (e.g., due to abiotic and biotic stress), but also plant development over a longer time range (up to several weeks).

In summary, this new phenotyping platform represents an important step in bridging lab and field phenotyping experiments. Moreover, it allows to link bottom-up and bottom-down approaches in the systematic investigation of the gas exchange between plants and the atmosphere.

## Methodology

### Setup

**Fig. 1:**
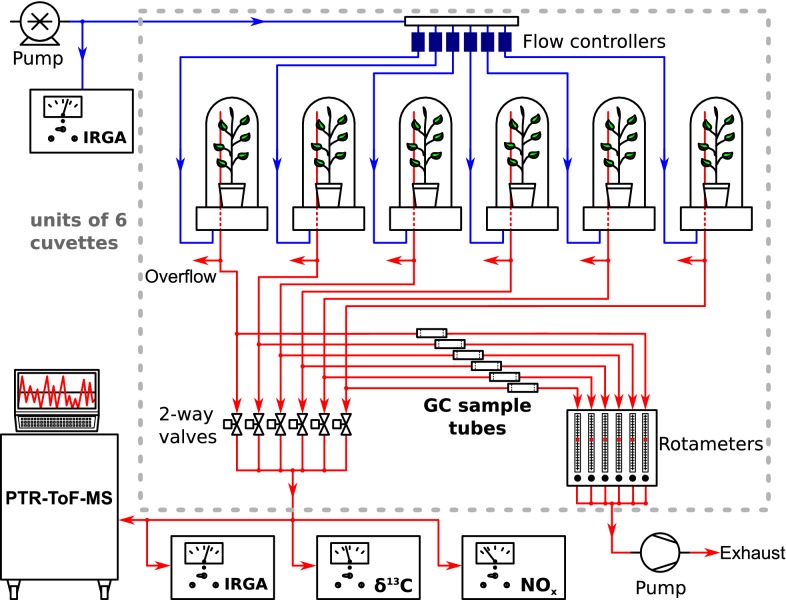
Schematic of the multiple cuvette system A rotary vane compressor pumps air from the surrounding phytotron chamber into the single cuvettes (blue lines). These are installed in units of 6 cuvettes on different tables. The cuvette outlets (red lines) are connected to (optional) GC sample tubes and different gas analysers, which sample from one cuvette at a time (controlled by a multiplexing system)

Figure 1 illustrates the setup of the VOC-SCREEN platform. The central element, the cuvettes, consist of a gas tight cylindrical base made of stainless steel and a glass cover. The base contains feed-throughs for gas and irrigation tubing, and electrical connections. Its top cover comprises a central hole for the installation of the plant pots. A fitting mechanism enables to use pots of different size, of up to 13 cm diameter. From the cuvette base, the supplied air is flushed into the cuvette air space via a circular system of dozens of small inlet holes. Test measurements with colour cartridges installed in front of the inlet have shown that this air distribution system enables good turbulent air mixing inside the cuvette. We refrained, therefore, from installing additional fans.

Under regular working conditions the soil is not separated from the above-ground area, unless it is covered and sealed with, e.g., Teflon^®^ foil.

A cylindrical Duran^®^ glass with a flattened semi-spherical top of 60 cm total height and 29 cm inner diameter (total volume of $\sim 40$l) constitutes the top cover of the cuvettes. These glass bulbs are clamped to the base with inert Viton rings sealing the joint.

Each cuvette is equipped with a combined air temperature and humidity sensor (DKRF400, Driesen + Kern GmbH, Bad Bramstedt, Germany), and a combined soil temperature and humidity sensor (5TM soil moisture and temperature sensor, Decagon Devices Inc., Pullman, WA, USA). In order to minimize radiative heating, the air temperature and humidity sensors are encased with a radiation protection (TR350, Driesen + Kern GmbH, Bad Bramstedt, Germany).

All tubing to and from the cuvettes is made of either PTFE or PFA Teflon^®^ in order to minimize deposition and reactions of VOCs on the tubing surfaces. Cuvettes are fed with particle and charcoal-filtered air drawn from the phytotron chamber via an oil-free rotary vane compressor (DLT 40, Gardner Denver Schopfheim GmbH, Schopfheim, Germany). As the maximum outlet pressure of the pump is $\le 1$ bar(g), under typical operation conditions of the phytotron chamber, the absolute humidity of the air is not changed by the pump. Cuvette inlet flows can be regulated in a range of 0–50 l min$^{-1}$ via mass flow controllers (Mass Stream D-6361, M+W Instruments GmbH, Leonhardsbuch, Germany). The cuvette outlet lines are connected via a T-piece to (optional) gas chromatography (GC) sample tubes and to a multiplexing system consisting of 2-way solenoid valves (cf. Fig. 1). When installed, the GC sample tubes are continuously flushed with air from a specific cuvette. One the other hand, the different multiplexing valves are opened one at a time, allowing the air from the specific cuvette to be directed to an online mass spectrometer (PTR-ToF-MS, Ionicon Analytik GmbH, Innsbruck, Austria; see below) and an Infra-Red Gas Analyser (IRGA; LI-840A, LI-COR Biosciences, Lincoln, Nebraska; measuring $\text{CO}_{2}$ and $\text{H}_{2}\text{O}$ concentrations) connected in parallel. If required, further measurement devices such as a $\delta {^{13}{\hbox{C}}}$ sensor, ${{\mathrm{NO}}_{\mathrm{x}}}$ ($\equiv {\text{NO}}+{\text{NO}}_{2}$) analyser, etc., can be installed.

An additional IRGA is used to measure the absolute $\text{CO}_{2}$ and $\text{H}_{2}\text{O}$ concentrations at the inlet of the cuvettes.

The cuvettes must be operated in a slight overpressure mode in order to prevent air leaking from the surrounding into the cuvettes. Cuvette inlet flows are therefore set higher than the flows to all the sensors and GC sample tubes, necessitating an overflow at each cuvette outlet.

Each six cuvettes are combined to an unit and placed onto tables for easier access. For each of this units a separate water supply is available, allowing to irrigate all plants of one unit with a specific amount of water via a distribution system.

The whole cuvette system, including the inlet flows, irrigation, valves and individual sensors, is controlled via a custom LabView^®^ based software. This software allows to display and save all acquired data at user defined intervals and is responsible for the switching of the 2-way multiplexing valves in a timed manner, typically in 5 min intervals. Accordingly, the sensors downstream the cuvettes sequentially sample air from different cuvettes.

### General workflow

**Fig. 2:**
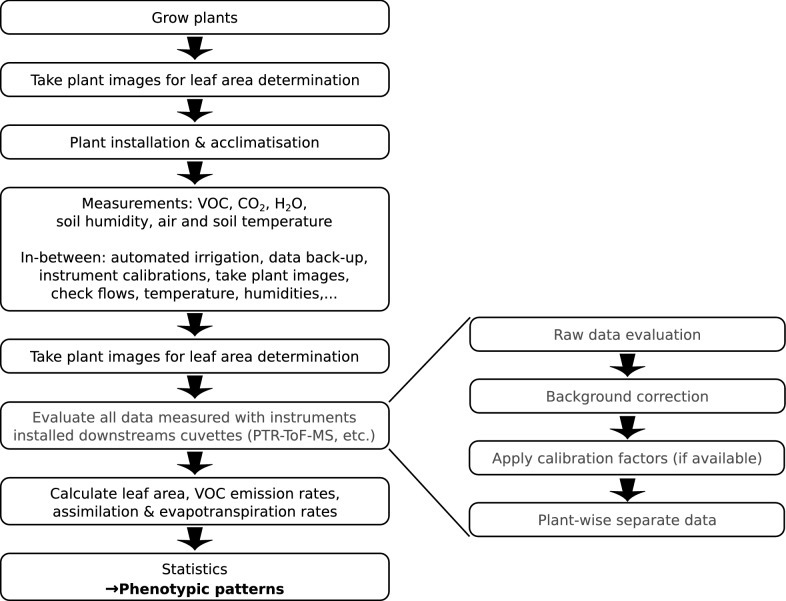
Workflow A phenotyping experiment with the VOC-SCREEN facility requires several working steps. These include the growing of the plants, leaf area determination, the actual measurement and data processing. With more different genotypes investigated, it might be necessary to apply multivariate statistics in order to detract phenotypic patterns

The platform is designed to determine phenotypic markers of different plants or plant varieties at different treatments. Here, the phenotypic traits are basically differences in VOC emission or uptake rates, net $\text{CO}_{2}$ assimilation and transpiration rates. To obtain and quantify these traits several different working steps prior, during and after a phenotyping experiment are needed (see Fig. 2). In general, before and after an experiment the leaf area of the plants investigated must be determined. For leaf area determination, pictures of the plants have to be taken outside of the cuvettes (see below). Afterwards, the plants are installed into the cuvettes and are allowed to acclimatize to the new environment. Typically one to two days after plant installation the actual measurements start. During the experiments, plants have to be irrigated, flows and environmental parameters have to be checked, data must be backed-up and instruments have to be calibrated. Depending on the length of an experiment and the plant’s growth rate additional leaf area measurements might be necessary. PTR-ToF-MS data processing after an experiment includes the evaluation of the raw data, background correction, applications of sensitivity factors to the calculated signals (in counts per second, cps) and finally the cuvette-wise data separation and normalization of the signals to the plant leaf area and cuvette inlet flow (see below). Assimilation (net $\text{CO}_{2}$ ecosystem exchange) and transpiration rates of the individual plants are calculated from the measured $\text{CO}_{2}$ and ${\text{H}}_{2}{\text{O}}$ concentrations at the inlet and outlet of the cuvettes, and the cuvette temperature. Eventually, multivariate statistics might be necessary to actually differentiate different phenotypes.

In the following sections the individual steps to be performed during a cuvette experiment are described in more detail.

### VOC measurements with PTR-ToF-MS

The primary focus of our new plant phenotyping platform lies on the VOC emissions of the plants investigated, ranging from low-emitting species such as crops to terpenoid-rich aroma plants and trees [29]. These emissions are measured on-line using a PTR-ToF-MS [23]. Basic principle of this instrument is the soft ionization of VOCs via proton transfer from primary ${\hbox{H}}_{3}{\hbox{O}}^{+}$ ions according to the following reaction scheme:

1 $$\begin{aligned} {\hbox{H}}_{3}{\hbox{O}}^{+} + {\hbox{VOC}} \rightarrow {\hbox{VOCH}}^{+} + {\hbox{H}}_{2}{\hbox{O}} \end{aligned}$$

This reaction is exothermic, if the proton affinity (PA) of the VOC is higher than that of water (691 kJ mol$^{-1}$ [30]), which is the case for most naturally occurring VOCs apart from some short-chained hydrocarbons [31]. The PTR-ToF-MS used in this study is equipped with a Selective Reagent Ionization (SRI) unit, by which alternative primary ions can be generated, such as ${\text{O}}^{+}_{2}$ or ${\text{NO}}^{+}$.

${\text{O}}^{+}_{2}$ reacts primarily via an electron transfer route [32–35]:

2 $$\begin{aligned} {\hbox{O}}_{2}^{+} + {\hbox{VOC}} \rightarrow {\hbox{VOC}}^{+} + {\hbox{O}}_{2} \end{aligned}$$

This reaction is exothermic for all VOCs having an ionization energy (IE) lower than the recombination energy of $\text{O}^{+}_{2}$ ($\sim 12.07$ eV), which is the case for almost all VOCs [31]. Operating the PTR-ToF-MS in $\text{O}^{+}_{2}$ primary ion mode thus does also allow to ionize the important plant signalling molecule ethene, which cannot be ionized via proton transfer ($\mathrm{IE_{ethene}} =10.51$ eV [31], $\mathrm{PA_{ethene}} =680.5$ kJ mol$^{1}$ [30]). However, as the energy transfer during an electron transfer reaction from $\text{O}^{+}_{2}$ to a VOC molecule often is fairly large, this ionization mechanism yields to a higher fragmentation rate than proton transfer [32–35].

When the PTR-ToF-MS is operated using $\text{NO}^{+}$ ($\mathrm{IE_{NO}} =9.26$ eV [31]) as primary ion, besides electron transfer other reaction mechanisms can also take place:

- *Hydride ion transfer*: in this case, an ${\hbox{H}^{-}}$ ion is detracted from the VOC:
  3 $$\begin{aligned} \hbox{NO}^{+} + \hbox{VOC}-\hbox{H} \rightarrow \hbox{VOC}^{+} + \hbox{HNO} \end{aligned}$$
  This reaction is important for (mono-) aldehydes and primary and secondary alcohols [32, 33].
- *Hydroxide ion transfer*: in this reaction an $\hbox{OH}^{-}$ group is detracted:
  4 $$\begin{aligned} \hbox{NO}^{+} + \hbox{VOC}-\hbox{OH} \rightarrow \hbox{VOC}^{+} + \hbox{HONO} \end{aligned}$$
  This reaction is relevant for tertiary alcohols (to a minor extend for primary, secondary and unsaturated alcohols) [33, 36] and (short chained) acids [34].
- *Clustering reactions*: depending on the operation conditions (i.e., electric field strength, pressure, temperature inside the PTR-ToF-MS’ reaction chamber), $\text{NO}^{+}$ ions can also form clusters with VOCs:
  5 $$\begin{aligned} \hbox{NO}^{+} + \hbox{R} + \hbox{M} \rightarrow \hbox{R}\,\bullet \,\hbox{NO}^{+} + \hbox{M} ,\quad \hbox{M}=\hbox{N}_{2}, \hbox{O}_2,\dots \end{aligned}$$
  This reaction is important for ketones [33], acids (competing with hydroxide ion transfer) and esters [34].

Since specific compound classes react via different reaction channels with $\text{NO}^{+}$, operating the PTR-ToF-MS in $\text{NO}^{+}$ reagent ion mode allows to differentiate several isomeric compounds, such as the ketone methyl vinyl ketone and the aldehyde methacrolein (both $\hbox{C}_{4}\hbox{H}_{6}\hbox{O}$) [37]. This is a substantial benefit in respect to the $\hbox{H}_{3}\hbox{O}^{+}$ reagent ion mode. On the downside, the different reaction channels in $\text{NO}^{+}$ reagent ion mode can sometimes occur simultaneously for the same compound [32–34], eventually leading to many more peaks in the mass spectrum and possibly complicating data interpretation.

### VOC data analysis

For the PTR-ToF-MS raw data analysis we use the routines and methods described by Müller et al. [38]. Data post-processing is performed in Matlab^®^.

After an experiment, the data from sensors installed downstream of the cuvettes has to be separated cuvette-wise. Thereby we have to consider the gas exchange time of the tubing from the cuvette outlets to the sensors. Data acquired within this specific lag time after switching the 2-way valves is omitted.

The HMGU phytotron chambers are run with preprocessed ambient air (particle and charcoal-filtered outside air). To correct for unavoidable contaminants in the cuvette air, e.g. from outgassing plastic parts of the sensors, measured VOC concentrations have to be background corrected. To this end, during every experiment some cuvettes remain empty (i.e., no plant at all or pots with only soil installed). Data of these background cuvettes is afterwards interpolated with a cubic spline function and subtracted from that of all other cuvettes.

### VOC emission rate calculation

The total quantity $q_{\mathrm{i}}(t)$ and concentration $c_{\mathrm{i}}(t)=q_{\mathrm{i}}(t)/V$, respectively, of a compound $\mathrm{i}$ at a time point *t* in the air volume *V* enclosed by the cuvette depend on different factors: the plant net emission rate $E_{\mathrm{i}}(t)$ (i.e., emission minus uptake rate) and its potential deposition on plant and cuvette surfaces. Moreover, its concentration is influenced by the amount entering and leaving the cuvette, driven by the air flow. Neglecting deposition on plant and cuvette surfaces, the temporal change of the total quantity of a compound $\mathrm{i}$ inside the cuvette can therefore be described with the following first order differential equation:

6 $$\begin{aligned} \dfrac{d}{dt}q_{\mathrm{i}}(t)= E_{\mathrm{i}}(t)\cdot A_{\mathrm{leaf}} + F_{\mathrm{in}} \cdot \dfrac{q_{\mathrm{i,in}}(t)}{V} - F_{\mathrm{out}} \cdot \dfrac{q_{\mathrm{i,out}}(t)}{V} \end{aligned}$$

with $F_{\mathrm{in}}$ and $F_{\mathrm{out}}$ the cuvette in- and outflow, and $q_{\mathrm{i,in}}$ and $q_{\mathrm{i,out}}$ the total quantity of the compound in the air entering or leaving the cuvette, respectively. $A_{\mathrm{leaf}}$ denotes the enclosed leaf area. Neglecting $\text{CO}_{2}$ assimilation (which is generally counterbalanced by the emission of oxygen) and water transpiration the cuvette inlet air flow equals the outlet flow (this is reasonable when the cuvette gas exchange is fast and transpired water adds little to the total flow) and therefore Eq. (6) can be simplified to

7 $$\begin{aligned} \dfrac{d}{dt}q_{\mathrm{i}}(t)= E_{\mathrm{i}}(t)\cdot A_{\mathrm{leaf}} + F_{\mathrm{in}} \cdot \dfrac{q_{\mathrm{i,in}}(t)-q_{\mathrm{i,out}}(t)}{V} \end{aligned}$$

Here we assume that the turbulent mixing in the cuvette is fast and therefore the concentration of any compound is homogeneous throughout the cuvette. Depending on the emission function $E_{\mathrm{i}}(t)$, $q_{\mathrm{i}}(t)$ can be calculated as algebraic function or might be calculated numerically.

Under steady state conditions $\frac{d}{dt}q_{\mathrm{i}}(t)=0$ and therefore Eq. (6) simplifies to the well-known formula [39, 40]

8 $$\begin{aligned} E_{\mathrm{i}}=\dfrac{F_{\mathrm{in}}}{A_{\mathrm{leaf}}} \cdot \dfrac{q_{\mathrm{i,out}}(t)-q_{\mathrm{i,in}}(t)}{V}= \dfrac{F_{\mathrm{in}}}{A_{\mathrm{leaf}}} \cdot ( c_{\mathrm{i,out}}-c_{\mathrm{i,in}}) \end{aligned}$$

$c_{\mathrm{i,in}}$ and $c_{\mathrm{i,out}}$ can be calculated from the calibrated signals measured with the PTR-ToF-MS.

### Net $\text{CO}_{2}$ assimilation and transpiration rate calculations

Net $\text{CO}_{2}$ assimilation (gross net assimilation minus photo-respiration and mitochondrial respiration) and transpiration rates of the plants can be estimated from the differences in $\text{CO}_{2}$ concentrations and absolute humidity between cuvette inlets and outlets, and the calculated leaf area [41]. The leaf temperature can be approximated using the air temperature within the cuvettes. This is justified, as also the rates are calculated as an integral over the whole cuvette, where light conditions and consequently photosynthesis are non-uniformly distributed over the entire plant canopy (with possibly shaded leaves). Since in our cuvettes the soil space is normally not separated from the above-ground area, soil respiration and evapotranspiration contribute to the corresponding $\text{CO}_{2}$ and $\text{H}_{2}\text{O}$ concentrations measured at the cuvette outlets. We therefore correct the absolute humidity and $\text{CO}_{2}$ levels at the plant cuvette outlets for the values measured from reference cuvettes containing pots with bare soil. Nonetheless, the calculated net $\text{CO}_{2}$ assimilation and transpiration rates might still be error-prone, due to the altered microbial activity in bare soil lacking plant mycorrhiza.

### Leaf area analysis

The calculation of, e.g., VOC emission or net $\text{CO}_{2}$ assimilation rates requires to know the leaf area of the investigated plants. For short experiments or slowly growing plants, leaves can be harvested and scanned after the experiment. The leaf area, determined using free tools like the Leaf Area Calculator (https://sites.google.com/site/ptrtof/file-cabinet) or ImageJ (http://imagej.net) can then be considered representative for the whole experiment. In all other cases, though, the leaf area must be analysed at different time points during the experiment (requiring to remove the plants from the cuvettes for a short time) or at least prior and after the experiment [10, 42].

**Fig. 3:**
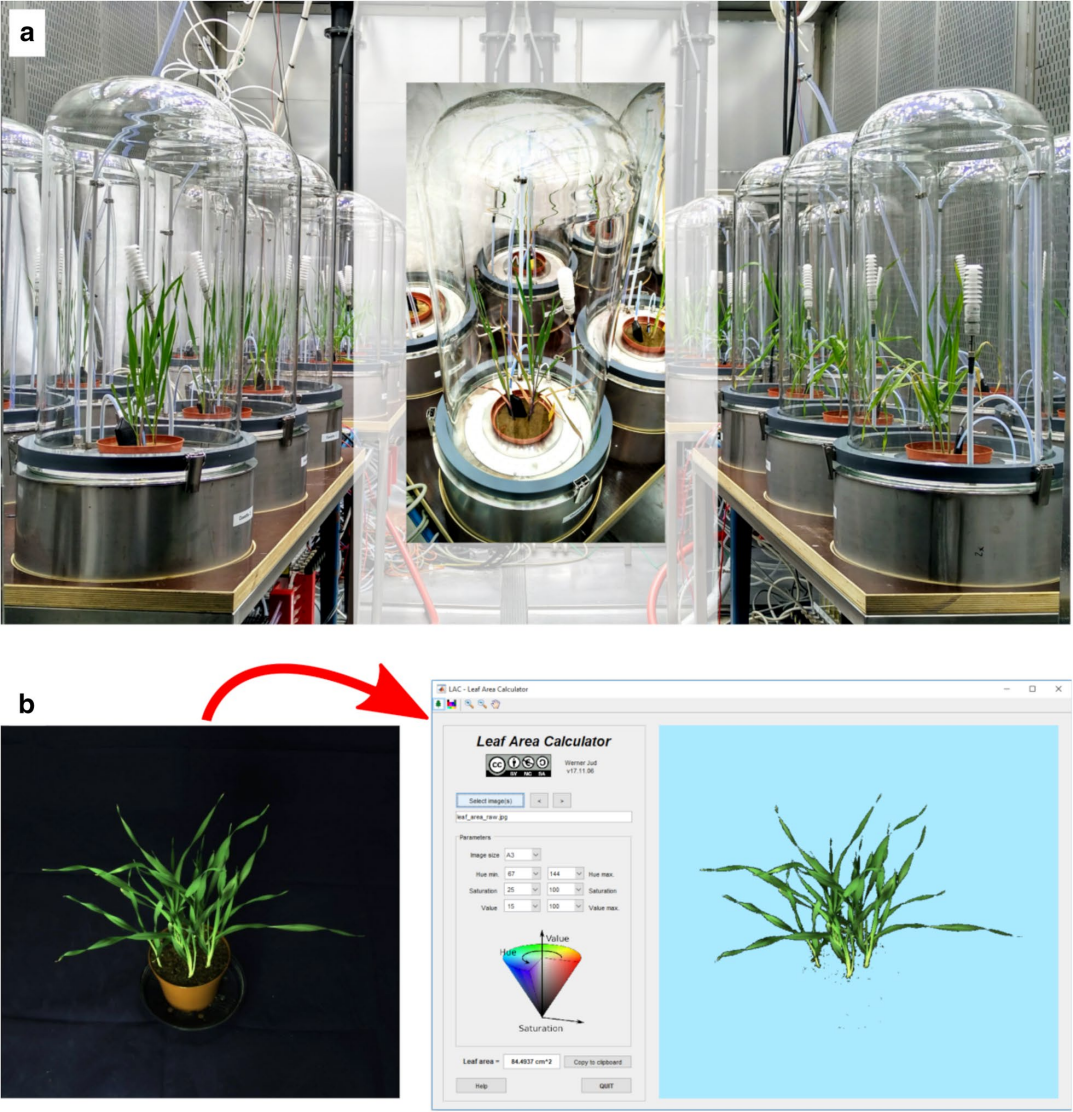
Photo of the cuvettes and leaf area determination. **a** The VOC-SCREEN platform installed in a phytotron chamber of the Helmholtz Zentrum München. **b** Plant images taken before and after a phenotyping experiment are fed to the Leaf Area Calculator program to infer the leaf area of the plants investigated. Eventually the leaf areas are used to calculate plant emission and uptake rates

Similar to Hartmann et al. [42] we take plant pictures from different angles (nine from front view, nine from a $45^{\circ }$ angle from above) in a light tent. A stepper motor allows to rotate the plants in front of an adequate image background (e.g., blue curtain). Subsequently, using the Leaf Area Calculator, all pixels of the image representing plant leaves (usually greenish pixels), are calculated. From calibration measurements (measured, exact leaf area vs. extracted pixles) using the same plant type we can eventually infer the actual leaf area (see Fig. 3b). This procedure is typically repeated every week; leaf areas in-between these time points are interpolated using a cubic spline. In the case of an unusual leaf growth or senescence, e.g., in the case of pathogen infection or drought periods, more frequent leaf area measurements might be necessary.

### Characterisation of the cuvette system

In order to characterise the performance of the new cuvette system a set of experiments was conducted, which will be described in the next sections.

#### Cuvette dynamics in response to changes in ambient light, air temperature and humidity

First of all we tested the responsiveness of the cuvettes’ microclimate to varying ambient conditions in the phytotron chamber. Nine of the cuvettes were fitted with barley plants while the other 15 cuvettes remained empty. In the course of two weeks we then continuously altered relative humidity, temperature and PAR within the phytotron chamber. Additionally, the cuvette inlet flows were changed in three steps from 10–20 l min$^{-1}$.

#### Case example: VOC emissions of barley treated with the elicitor benzothiadiazole (BTH)

To demonstrate the capabilities of the VOC-SCREEN platform, we performed an experiment in which different spring barley varieties (*Hordeum vulgare* ’Barke’, ’Golden Promise’ and ’Morex’) were treated with a solution of the elicitor benzo-(1,2,3)-thiadiazole-7-carbothioic acid-S-methyl ester (BTH, $\hbox{C}_{8}\hbox{H}_{6}\hbox{N}_{2}\hbox{OS}_{2}$). The BTH treatment simulates infestation of the plants by a biotrophic pathogen (see below). Each nine seeds were planted in pots of 13 cm diameter, containing a soil mix of 40% Hawita Fruhstorfer Einheitserde type LD 80, 40% loam, 10% sand and 10% vermiculite. Prior to experiment the plants were grown for three weeks in phytotron chambers under the same environmental conditions as during the later experiment (12/12-h photoperiod, 300–350 $\upmu \hbox{mol m}^{-2}\hbox{ s}^{-1}$ photosynthetically active radiation at the canopy level; temperature and relative humidity were set to 15 °C/18 °C and 57%/45%, respectively, during night/day; $\text{CO}_{2}$ concentration was ambient at $\sim$400 ppm).

The 1 mM BTH solution was prepared by dissolving 42 mg of the pure BTH (trade name Acibenzolar-S-methyl, Merck KGaA, Darmstadt, Germany) in 10 ml of HPLC grade acetone (Carl Roth GmbH, Karlsruhe, Germany). Afterwards the solution was poured in 20 ml of double distilled water, together with 100 $\upmu \text{l}$ of Tween-20 (Merck KGaA, Darmstadt, Germany), and stirred for 10 min. Eventually the solution was poured into 180 ml of double distilled water and stirred further for about 16 h. While stirring, the glass jar remained open to allow for evaporation of acetone. For the mock treatment the corresponding solution without BTH was prepared.

Seven pots of each barley variety were installed in the cuvettes at day -1 of the experiment and allowed to adapt to the cuvette microclimate for one day. In the remaining three cuvettes pots with bare soil were installed. These cuvettes were used for the background measurements. Since barley plants are low VOC emitters [43], the cuvette inlet flow was set as low as possible (6 $\text{l min}^{-1}$) in order the minimize dilution of plant VOC emissions. Gas exchange measurements were performed using two IRGAs connected to the inlet and outlet of the cuvette system, respectively, and a PTR-ToF-MS connected only to the outlet of the cuvettes (see "Setup" section). Prior (and after) the experiment the leaf area of the barley plants was estimated using the routines described above.

At day 0 of the experiment four plant pots of each variety were treated with BTH by spraying the solution on the leaves using a pump atomizer. Three plant pots of each variety were treated with the mock solution in the same way. The experiment lasted until day 6, when induced emissions (measured online with the PTR-ToF-MS) apparently had levelled off.

The whole experiment was performed twice using the same BTH solution in order to avoid concentration variations due to systematic errors. In the meanwhile, the solution was stored in the dark to prevent possible photo-dissociation [44].

In order to quantify possible photolysis reactions of BTH during the experiments, we conducted additional tests with plant mock-ups made of stainless steel wool. Six cuvettes were equipped with pots containing bare soil and the plant mock-ups; three of the mock-ups were sprayed with the BTH solution similar to the plant treatment, while the remaining three mock-ups were not treated at all. Afterwards, the cuvettes were exposed to the same climate and light conditions as used in the plant experiment.

## Results and discussion

### Cuvette dynamics in response to changes in ambient light, air temperature and humidity

As plant VOC emissions are generally very low ($\sim \hbox{nmol m}^{-2}\,\hbox{s}^{-1}$ range) compared to, e.g., $\text{CO}_{2}$ uptake ($\sim \upmu \hbox{mol m}^{-2}\,\hbox{s}^{-1}$ range) or water transpiration ($\sim \hbox{mmol m}^{-2}\,\hbox{s}^{-1}$ range), it is advisable to keep the cuvette inlet flows $F_{\mathrm{in}}$ low in order to minimize dilution. However, in practice depending on the plant type/species investigated, the plant water status, light conditions, etc., this could result in $\text{CO}_{2}$ deficiency, condensation of transpired water, or excessive heating inside the cuvettes. Condensation of water vapour must be avoided, as otherwise signals of water soluble VOCs, such as methanol, might be distorted. The cuvette inlet flow setting should also allow having a possibly high sample flow to the PTR-ToF-MS in order to minimize deposition of larger and polar, semi-volatile compounds at the cuvette and tubing surfaces [45]. As a high cuvette inlet flow decreases the concentration of VOCs to be measured in the outlet air, the only way to improve the performance of such a cuvette system is to increase the sensitivity and thus the limit of detection (LOD) of the PTR-ToF-MS. To this end, we used a PTR-ToF-MS instrument equipped with an ion funnel between ionisation chamber and mass spectrometer. This improves its sensitivity compared with a traditional PTR-ToF-MS by a factor of $\gtrsim 5$. Nevertheless, in practice in each experiment a good compromise has to be found by keeping the cuvette inlet flows as small as possible but as large as necessary in order to avoid perturbations.

The cuvette inlet flow and the cuvette volume *V* define two important parameters of a cuvette system: the characteristic time constant $\tau$ and its inverse, the exchange rate $1/\tau$ of the cuvette system:

9 $$\begin{aligned} \tau =\dfrac{V}{F_{\mathrm{in}}} \end{aligned}$$

With a cuvette inlet flow of 10 $\text{l min}^{-1}$/15 $\text{l min}^{-1}$/20 $\text{l min}^{-1}$ and a cuvette volume of about 40 l, it takes 20 min/13.3 min/8 min ($\equiv 5\tau$) until the air inside one of the cuvettes is exchanged to more than 99% [46]. As a flow-through system the cuvettes can be considered as low-pass filter system; fast plant emission bursts inside the cuvette are “washed-out” by the entering air. To capture VOC emission patterns in a most realistic way it is therefore recommended to use a possibly small cuvette volume to obtain a $\tau$ considerably lower than the fastest emissions expected [46]. To accommodate this requirement, in future glass covers of different size will be available for the cuvettes of the VOC-SCREEN platform, which can be exchanged depending on the size/biomass of the plant investigated.

**Fig. 4:**
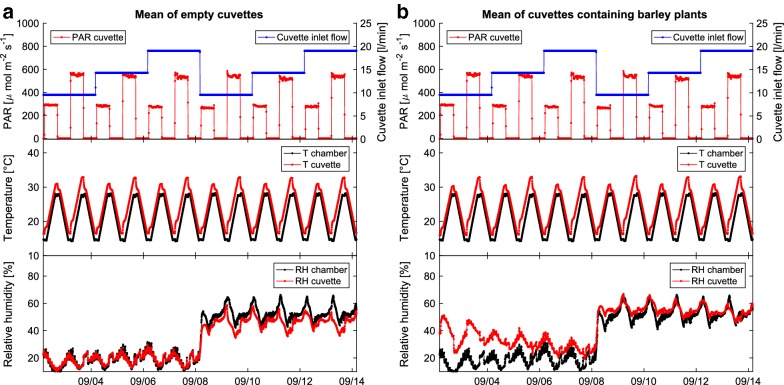
Cuvette temperature and humidity dynamics Air temperature and relative humidity dynamics inside **a** empty cuvettes or **b** cuvettes with barley pots installed when varying ambient parameters and cuvette inlet flow. During this experiment temperature and relative humidity in the phytotron chamber, photochemical active radiation (PAR) and cuvette inlet flows were altered. High PAR caused radiative heating inside the cuvettes, which was almost independent from the cuvette inlet flow investigated (10–20 $\text{l min}^{-1}$). Plant evaporation increased the relative humidity inside the cuvettes especially when having dry air pumped from the phytotron chamber into the cuvettes. Under the tested conditions the presence of plants in the cuvettes had little to no effect on the temperature dynamics [data is mean of 15 empty cuvettes (**a**) and 9 plant cuvettes (**b**)]

In test measurements with empty cuvettes and cuvettes containing pots with barley we investigated the temperature and humidity dynamics inside the cuvette relative to the temperature and humidity in the phytotron chamber housing of the cuvette system (see “Methodology” section). During this experiment, also the cuvette inlet air flows and the light conditions were changed. Figure 4 illustrates the results of this physical characterisation. Apparently, when altering the temperature of the chamber, the cuvette temperature changed at a time scale $> \tau$ due to the inertia of the system. This inertia was caused by the large heat capacity of the cuvette’s metallic base and the glass cover.

In general, when the cuvettes were illuminated, the temperatures inside were higher than in the surrounding climate chamber. This behaviour was related mainly to the radiative heating caused by illumination, even though major parts of infra-red radiation were shielded off by a water filter underneath the lamps of the phytotron chamber [26, 27, 47].

As shown in Fig. 4, the temperature differences between cuvettes and the surrounding air were almost independent on the cuvette inlet flow range investigated. They were rather depending mainly on the intensity of the incident radiation. Under full light ($\sim 600\,\upmu \hbox{mol\,m}^{-2}\hbox{ s}^{-1}$ within the cuvette) the temperatures inside the cuvettes were up to 5 $^{\circ }$C higher than in the surrounding chamber. Moreover, this experiment suggested that this difference was more or less independent from the presence of a (barley) plant inside the cuvette, that potentially could have led to a temperature reduction due to evaporative cooling.

This observation can be verified theoretically. Under steady state conditions, in a (very) rough estimate all the energy needed for plant transpiration, i.e., the enthalpy of vaporization $H_{\mathrm{v}}$ of an amount *n* of water with mass *m*, equals the energy removed from the inflowing air to reach the measured steady-state temperature (assuming fast turbulent mixing inside the cuvette). The air is thus cooled by $\Delta T$, equalling the temperature difference of cuvettes equipped with plants in respect to empty cuvettes:

10 $$\begin{aligned} \Delta Q = n H_{\mathrm{v}} = m \ c_p \ \Delta T \end{aligned}$$

with the specific heat capacity at constant pressure $c_{\mathrm{p}}$.

Differentiating the middle and the right term of Eq. 10 we get

11 $$\begin{aligned} \dfrac{dn}{dt} H_{\mathrm{v}} = \dfrac{dm}{dt} \ c_{\mathrm{p}} \ \Delta T \end{aligned}$$

With the transpiration rate $E_{\mathrm{s}}$, leaf area *A*, air density $\rho _{\mathrm{air}}$ and cuvette inlet flow $F_{\mathrm{in}}$ we can write $dn= E_{\mathrm{s}}\ A\ dt$ and $dm=\rho _{\mathrm{air}}\ F_{\mathrm{in}}\ dt$.

Equation (11) can thus be rewritten as

12 $$\begin{aligned} E_{\mathrm{s}} \ A\ H_{\mathrm{v}} = \rho _{\mathrm{air}}\ F_{\mathrm{in}} \ c_{\mathrm{p}} \ \Delta T \Longrightarrow \end{aligned}$$

13 $$\begin{aligned} \Delta T = \dfrac{E_{\mathrm{s}} \ A\ H_{\mathrm{v}}}{\rho _{\mathrm{air}}\ F_{\mathrm{in}} \ c_{\mathrm{p}}} \end{aligned}$$

With a plant transpiration rate of 3 $\upmu \hbox{mol m}^{-2}\hbox{ s}^{-1}$, an enclosed leaf area of 0.5 $\hbox{m}^{2}$, cuvette inlet flow of 10 $\text{l min}^{-1}$, a calculated air density of $1.01\,\hbox{kg}^{3}\,\hbox{m}^{-1}$ (at an air pressure of 96,000 Pa, air temperature of 298.15 K), specific heat capacity $c_{\mathrm{p}}=1106\,\hbox{J kg}^{-1}\hbox{ K}^{-1}$ (at an air relative humidity of 80%, air temperature of 298.15 K) and an enthalpy of vaporization of water $H_{\mathrm{v}} =43.99$ kJ mol^−1^ (calculated with the Clausius–Clapeyron equation) we calculated a temperature difference $\Delta T$ of 0.35 K (0.18 K at a cuvette inlet flow of 20 $\text{l min}^{-1}$). This value can be considered as maximum temperature difference between cuvettes operated at the given parameters with a barley plant installed or not installed. Under real conditions, additional factors might be considered, e.g., the darker surface of the leaves compared to the metallic surface of the cuvette base will absorb more radiation energy, counteracting the evaporative cooling mechanism.

Due to transpiration of the plants and water evaporation from the soil, the humidity inside the cuvettes was generally higher than in the surrounding air. At the beginning of this experiment the RH difference was about 20%. With higher inlet flow rates the RH difference between cuvette inside and outside decreased, down to $\sim 5\%$. We need to point out here that due to the unnatural climatic and light conditions (cf. Fig. 4) the plants were stressed and degrading over time, which led to lower transpiration and consequently lower RH differences, too.

In summary the characterization of the cuvette system has shown the expected behaviour: the infra-red part of the irradiation led to an increase in the cuvette temperature; especially under light conditions evapo-transpiration increased the relative humidity in an inlet-flow dependent manner. Surprisingly though, in the tested flow range the cuvette temperature at a given chamber temperature was almost independent from the cuvette inlet flow.

Practically, the temperature and RH difference between the inside of the cuvettes and the phytotron chambers must be taken into account when defining the climate parameters of a phenotyping experiment. The temperature offset of the phytotron chamber in respect to the desired temperatures inside the cuvettes has to be adjusted depending on the light intensity. The adjustment of the relative humidity is trickier, as the RH inside the cuvette is strongly dependent on the enclosed leaf area and the transpiration rate of the plants investigated. Furthermore, the latter can change when stress is applied to the plants. Humidity regulation is therefore rather complicated and might require continuous adaption throughout an experiment.

### Case study: VOC emissions of barley treated with the elicitor benzothiadiazole (BTH)

**Fig. 5:**
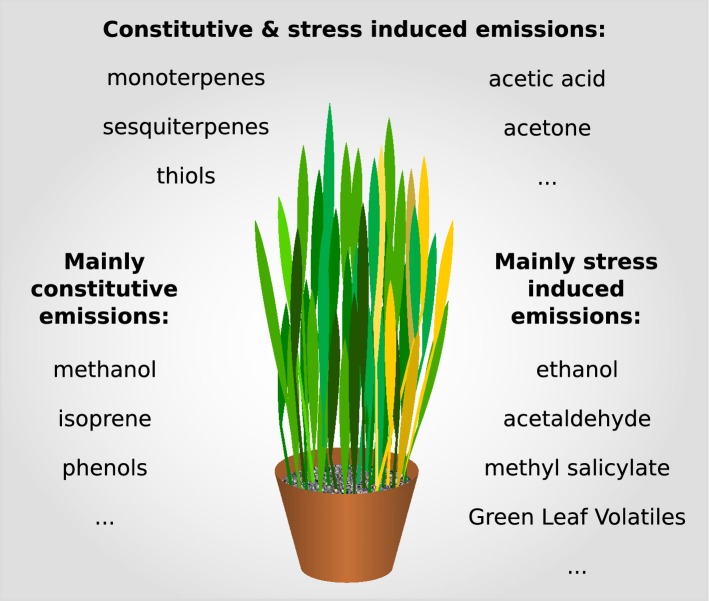
Plant VOC emissions Plant VOC emissions can be either constitutive or induced. Specific VOCs are attributable to either one or both of these emission classes

The profile of plant volatiles, aromatics, and essential oils (all together referred to as VOCs) is a complex phenotypic trait with tremendous metabolic diversity and source strength between different species [29], but also within a species [48]. VOCs can be produced constitutively as (by-) products of the regular plant metabolism or—as part of an inherent plant self-defence mechanism—in an induced manner (Fig. 5). Methanol for example is a typical plant growth marker and is formed during the demethylation of cell walls in the course of cell expansion [25]. Isoprene, the most abundant biogenic, non-methane VOC in the earth’s atmosphere with global emission rates of $\sim 1000\hbox{ Tg yr}^{-1}$ [49], is produced in the plastidic methyl-erythritol 4-phosphate (MEP) pathway in a light and temperature dependent fashion [50, 51]. Related terpenoid compounds—monoterpenes and sesquiterpenes—have both direct and indirect functions in plant defence. Due to their bioactivity [52] they can directly affect cell components and thus play a central role in the plant’s defence arsenal against herbivores [53], microbes [54, 55] and fungi [54]. On the other hand, in tritrophic interactions plants use terpenoid compounds as volatile signalling molecules to attract natural enemies of parasitoids [56, 57]. Moreover, terpenoids play a vital role as phytohormones [58] and as attractant for pollinators [59].

**Fig. 6:**
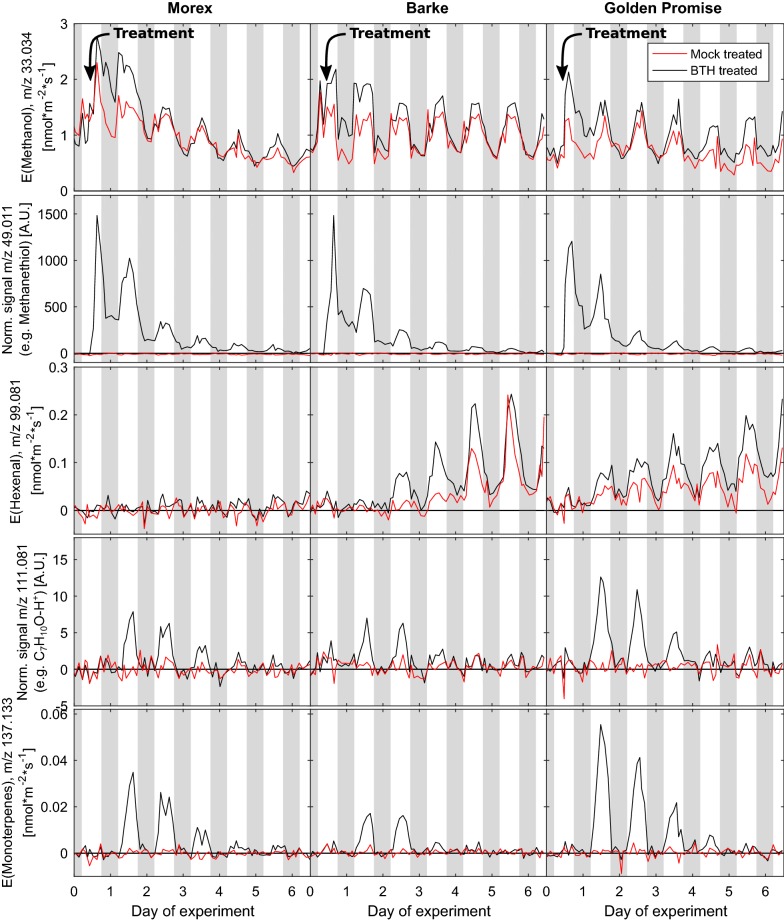
Barley variety dependent VOC emissions upon mock or BTH treatment Exemplary VOC emission rates of three different barley varieties (from left to right: Morex, Barke, Golden Promise) upon treatment with the elicitor BTH (black line) or mock treatment (red line). Distinct VOC emission patterns allow to differentiate the different genotypes. Methanol is a typical growth marker and was thus emitted by all plants. Hexenal is a typical stress marker and correlated with an apparent plant degradation towards the end of the experiment (no emission by Morex plants). BTH treatment induced the emission of methanethiol, an ion with sum formula $\hbox{C}_{7}\hbox{H}_{10}\hbox{O}$–$\hbox{H}^+$ and monoterpenes. (data are means of 8 biological replicates for each variety in the BTH treatment and of 6 biological replicates in the mock treatment; for compounds, where no PTR-ToF-MS sensitivity was available, the signals were only normalized to the plant leaf area)

An important class of compounds formed and released by plants during stress are the green leaf volatiles (GLVs) [60, 61]. Following mechanical wounding of plant tissue, lipase enzymes cause the release of linoleic (C18:2) and $\alpha$-linolenic (C18:3) acid from thylakoid membranes. These are degraded by lipoxygenase (LOX) enzymes. Further action of hydroperoxide-lyase (HPL), isomerisation factors, alcohol dehydrogenase (ADH) and alcohol acyltransferase eventually form the GLVs, comprising various volatile $\hbox{C}_{6}$ alcohols, aldehydes and esters thereof [60, 62].

The release of GLVs is not limited to mechanical wounding, but rather a common stress marker observed under a variety of different biotic and abiotic stresses. The emission of GLVs is typically one of the first signs of plant stress and often occurs long before the plants show any visible symptoms. This is why they are commonly used as non-invasive plant stress tracers [62–64].

**Fig. 7:**
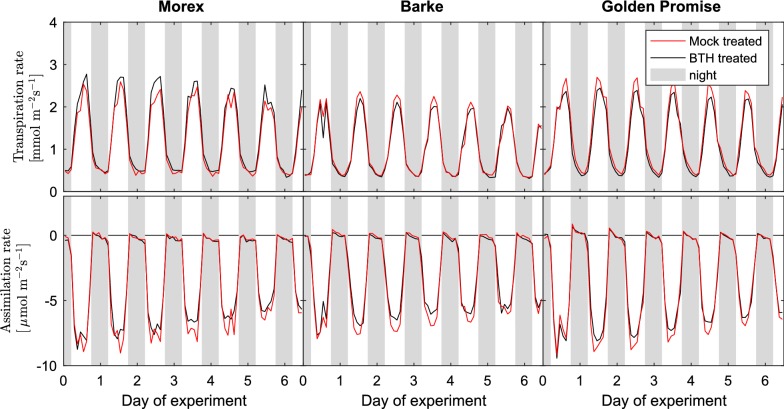
Barley variety dependent transpiration and net $\text{CO}_{2}$ assimilation rates upon mock or BTH treatment Transpiration rates (top) and net $\text{CO}_{2}$ assimilation rates of three different barley varieties (Morex, Barke, Golden Promise) upon treatment with the elicitor BTH (black line) or mock treatment (red line). (data are means of 8 biological replicates of each variety in the BTH treatment and of 6 biological replicates in the mock treatment)

Several VOCs, whose emission is induced under stress conditions, can be related to an either jasmonic acid (JA) or salicylic acid (SA) dependent pathway [64–66]. Both JA and SA are important signalling molecules within and in-between plants and activate a large set of measures to cope with the stressor. They play a key role in the immune response of plants.

The SA dependent pathway is activated mainly in response to biotrophic pathogens and eventually leads to a programmed cell death as an ultimate defence action. Conversely, the JA pathway is in general activated in response to necrotrophic pathogens, which might even benefit from cell death [65].

It has been shown that some compounds can mimic the role of JA or SA, eliciting a plant immune response themselves. These compounds, termed elicitors, can therefore be used to strengthen the plant resistance against specific pathogens [67, 68]. The activation of signal transduction pathways through elicitors can lead to the biosynthesis of phytoalexins, reinforcement of the plant cell walls, deposition of callose, synthesis of defence enzymes and accumulation of pathogenesis related (PR) proteins. In general, elicitors trigger the production of reactive oxygen species (ROS), which in turn lead to a hypersensitive response (HR). Upon pathogen infection, the HR causes a local cell death at the infection site to limit pathogen growth. Following HR, also uninfected, distal parts of the plant may develop resistance against further infection, a phenomenon known as systemic acquired resistance (SAR) [67]. This effect is associated with a local and systemic increase in SA levels [68].

One popular elicitor acting as SA agonist is benzo-(1,2,3)-thiadiazole-7-carbothioic acid S-methyl ester (BTH). BTH induces characteristic SAR-related responses similar to SA or pathogens, including the up-regulation of PR genes. BTH can be considered a functional analog of SA [68].

In the present proof-of-principle experiment we used BTH to induce stress-related VOC emissions in different spring barley varieties, i.e., Barke, Golden Promise and Morex. Herewith we aimed to demonstrate that the VOC-SCREEN platform can indeed be used to (1) continuously monitor the constitutive and induced emissions of 24 plants over a period of several days, and (2) to differentiate different plant varieties solely by their VOC emission patterns.

Barley is a rather low VOC-emitting species [43] and this proof-of-principle experiment can therefore also be regarded as kind of a benchmark test to demonstrate the overall capabilities of the new system. In fact, with the VOC-SCREEN platform any other kind of terrestrial plant (ranging from monocotyledonous to dicotyledonous crops, woody tree species and conifers) whose physical parameters match the size of the cuvettes can be tested for its constitutive and induced VOC emissions.

Thanks to the low detection limit of the instrument, in the spectral analysis of the PTR-ToF-MS raw data of this experiment we were able to identify 475 mass peaks corresponding to different mass to charge (m/z) ratios in the range of 1–315 u. Generally, these detected m/z ratios correspond to a large set of highly diverse (and in part also isomeric) compounds, their isotopes, and to a minor extend fragments and clusters with $\hbox{H}_{3}\hbox{O}^{+}$ ions thereof formed during the ionization reaction (cf. “Methodology” section) [23]. Most of the signals showed little to no variation in the course of a day when switching between the cuvettes and were consequently attributable to the background in the sample air and unavoidable instrument peaks. In order to facilitate data evaluation and to reduce the number of masses to be examined, the evaluated spectra were screened for m/z signals clearly demonstrating a cuvette and thus plant specific pattern. From the resulting list of $\sim 20$ m/z ratios, isotopes and known common fragments were discarded. In essence, nine different ion signals were attributed to different precursor molecules in barley and assigned to putative sum formulas:

- $$m/z\,33.034$$ ($$\hbox{CH}_{3}\hbox{OH}$$–$$\hbox{H}^{+}$$)
- $$m/z\,49.011$$ ($$\hbox{CH}_{3}\hbox{SH}$$–$$\hbox{H}^{+}$$)
- $$m/z\,63.027$$ ($$\hbox{C}_{2}\hbox{H}_{5}\hbox{SH}$$–$$\hbox{H}^{+}$$)
- $m/z\,83.086$ ($\hbox{C}_{6}\hbox{H}_{11}^{+}$, main fragment ion of $\hbox{C}_{6}\hbox{H}_{12}\hbox{O}$ [32, 61])
- $$m/z\,99.081$$ ($$\hbox{C}_{6}\hbox{H}_{10}\hbox{O}$$–$$\hbox{H}^{+}$$)
- $$m/z\,111.081$$ ($$\hbox{C}_{7}\hbox{H}_{10}\hbox{O}$$–$$\hbox{H}^{+}$$)
- $$m/z\,137.133$$ ($$\hbox{C}_{10}\hbox{H}_{16}$$–$$\hbox{H}^{+}$$)
- $$m/z\,143.107$$ ($$\hbox{C}_{8}\hbox{H}_{14}\hbox{O}_{2}$$–$$\hbox{H}^{+}$$)
- $$m/z\,153.128$$ ($$\hbox{C}_{10}\hbox{H}_{16}\hbox{O}$$–$$\hbox{H}^{+}$$)

Figure 6 exemplarily shows the time traces of some of the VOCs from different plant/treatment combinations following BTH treatment. Methanol emissions ($m/z\,33.034$) showed a typical light dependent diurnal variation representative for plant growth. The BTH treatment seemed to slightly enhance the methanol emission rates, although there was no apparent elevated growth rate of BTH treated plants.

There was a striking signal at $m/z\,49.011$, assigned to methanethiol ($\hbox{CH}_{4}\hbox{S}$–$\hbox{H}^{+}$), which was completely absent in mock treated plants. Methanethiol signals have also been observed upon infestation with barley mildew (*Blumeria graminis f. sp. Hordei*) of several other spring barley varieties (W. Jud, unpublished data). Methanethiol emissions appear to be a volatile marker for SA-induced stress responses. The emission of methanethiol followed a clear diurnal pattern pointing to a light-dependent biosynthetic process. In plants methanethiol is synthesized from bisulfide in a S-adenosyl-L-methionine-dependent reaction by a S-adenosyl-L-methionine:halide/bisulfide methyltransferase (EC 2.1.1.-) [69]. Whether this enzyme (or respective gene) became induced by BTH treatments must be analyzed in future studies.

Methanethiol signals, however, could also be caused by photo-dissociation of BTH at the surface of the plants [44]. To test for this possible additional methanthiol source, we installed plant mock-ups of stainless steel wool in the cuvettes and sprayed them with BTH solution similar as we did in the plant experiments (cf. “Methodology” section). When exposing the mock-ups to the phytotron chamber irradiation, only minor methanthiol production was observed ($\sim$ 10–20% of the signals measured in the plant experiment), indicating that the signal detected at $m/z\,49.011$ indeed originated to a major part from plant emissions.

The signal at $m/z\,99.081$ was attributed to hexenal isomers, which belong to the GLVs. The amount of these GLVs emitted by Barke and Golden Promise was increasing over time with a distinct diurnal variation. This observation is consistent with the observed yellowing of the leaves of these plant varieties towards the end of the experiment. We assume that these varieties might have responded to acetone, which was added to both the mock and BTH solution. Acetone was used to dissolve the BTH salt during solution preparation (cf. “Methodology” section). A potential role of acetone in the GLV emission is consistent with the fact that both mock *and* BTH treated Barke and Golden Promise plants showed these emissions. The Morex variety was apparently more tolerant to acetone.

Another—yet unidentified—compound appeared at $m/z\,111.081$ ($\hbox{C}_{7}\hbox{H}_{10}\hbox{O}$–$\hbox{H}^{+}$, e.g., norcamphor, a bicyclic ketone). The signals of this compound were clearly related to the BTH treatment and were highest from Golden Promise plants and lowest for Barke plants. To identify this compound further experiments involving trapping of the compound on absorption tubes followed by GC–MS analysis are necessary.

In addition, we observed the induction of monoterpene emissions ($m/z\,137.133$, $\hbox{C}_{10}\hbox{H}_{16}$–$\hbox{H}^+$). Monoterpene signals were detectable solely in the emission profiles of BTH treated plants and similar to the other VOC signals, showed a clear diurnal pattern. Again, for the identification of the emitted monoterpenes, GC cartridges could be installed in the VOC-SCREEN platform on specific days, when their corresponding signal in the PTR-ToF-MS is highest. This is essential for the separation of different isomeric monoterpenes, as hundreds and thousands of different isomers are known to be produced in the plant kingdom [53].

A recent study on Arabidopsis was able to link SA-dependent monoterpene emission to SAR of plants [55]. Our results support this theory, as BTH is eliciting a biotrophic reaction and in further consequence SAR.

Monoterpene emissions showed variety specific differences in intensity, similar to the $m/z\,111.081$ signals, thus indicating a somehow coordinated cultivar-specific response.

Besides VOC emission rates, the VOC-SCREEN platform delivers phenotype and treatment-specific information on the photosynthetic performance of the plants. Figure 7 displays the transpiration rates $E_S$ and net $\text{CO}_{2}$ assimilation rates *A* of the six plant-treatment combinations. The rates were very similar in all three cultivars and did not differ between BTH and mock treated plants. Only Barke plants exhibited a slightly lower transpiration rate. Nevertheless, the minimal differences in transpiration and net $\text{CO}_{2}$ assimilation rates are very unlikely to account for the observed difference in the VOC emissions.

## Conclusion

From a plant perspective, the synthesis of volatile compounds is a significant investment in carbon and energy, competing with other traits and thus potentially decreasing growth, carbon allocation, and yield. For this reason, traditional breeding and selection might have involuntarily decreased or altered VOC profiles in agricultural crops [70]. Plant VOCs, however, are important means of plant communication with their environment and as components of the plants’ defence arsenal. Plants use VOCs, e.g., to attract pollinators, as infochemicals “alarming” neighbouring plants or even distal parts of the same plant from pathogen and parasitoid or herbivore attack [51, 55, 59], or directly as antimicrobials [71, 72]. In respect of agricultural and even medical plants, VOCs are crucial parts of what makes up the taste and/or quality of the end product, e.g., fruits, legumes, herbs [73–76] or natural remedies [77, 78].

To some extent all plants emit VOCs both constitutively and in an induced manner (e.g., after pathogen or herbivore attack), which makes VOCs (intensity and patterns) an attractive target for the study on plant specific markers. Taken all together, there is good reasoning for volatilomic phenotyping of plants in the search for the most robust, sustainable and productive phenotype, better smell, or richness in bioactive compounds. Whilst measuring VOCs, the simultaneous measurement of $\text{CO}_{2}$ and $\text{H}_{2}\text{O}$ concentrations with the VOC-SCREEN platform even allows for phenotyping of plant photosynthetic performance and water use efficiency. These traits are important for plant breeding efforts with respect to improving drought tolerance and resource use efficiency [79, 80].

Compared to other phenotyping technologies, e.g., imaging approaches [7], volatilomics and gas exchange screening is a medium throughput approach. However, it is delivering dynamic information on the in vivo performance of very important plant traits under biotic and abiotic stress. A further extension to a higher throughput system would be simply a matter of costs and actual technological developments, with the sequential VOC sampling from the different cuvettes as main limiting factor. Currently, we perform VOC sampling from each cuvette every $\sim$ 2 h ($24 \times 5$  min). This is a sampling interval which we consider as upper limit in order to accurately track the diurnal variation in plant VOC emissions. Shortening the sample period of 5 min per cuvette is discouraged as this would result in the loss of more polar, sticky, and heavy compounds, whose transmission to the PTR-ToF-MS is bad, as long as no equilibrium between gas-phase and condensed phase at the tubing surface has established. By choosing an adequate inert tubing material (Teflon^®^ and PEEK) and heating the sample lines we try to minimize this effect.

An extension to a higher number of cuvettes would further decrease the cuvette-wise temporal resolution. If the VOC phenotyping would be limited to the light phase only, however, the number of plants measured in parallel could still be doubled by sampling from cuvettes installed in two separate climate chambers operated in opposite day/night mode (i.e., a time shift of 12 h). This approach could be reasonable, as VOC emissions typically follow a pronounced light- and temperature-dependent diurnal cycle with lowest emissions during the night [29, 81]. If this is not feasible, additional VOC sensors have to be implemented in order to maintain or even improve the temporal resolution of the recorded VOC emissions.

At the present, the PTR-ToF-MS represents the means of choice for online, highly sensitive VOC measurements. In combination with GC–MS analysis this allows gaining possibly high time-resolved and isomer specific information on the VOCs emitted by the sample plants. However, in future the development of new, cheaper and potentially more sensitive VOC detectors [82–87] in combination with machine-learning approaches will allow phenotyping of much more samples within the same time. The new system reflects the actual state-of-the art of plant gas exchange and online mass spectrometry, with its strength in phenotyping highly dynamic changes in the emissions and emission patterns of plants challenged by biotic and abiotic constraints.
